# Non-centrosomal MTs play a crucial role in organization of MT array in interphase fibroblasts

**DOI:** 10.3934/genet.2018.2.141

**Published:** 2018-03-28

**Authors:** Yekaterina Zvorykina, Anna Tvorogova, Aleena Gladkikh, Ivan Vorobjev

**Affiliations:** 1 Biology Department, M.V. Lomonosov Moscow State University, Moscow, Russia; 2 A.N. Belozersky Institute of Physico-Chemical Biology, M.V. Lomonosov Moscow State University, Moscow, Russia; 3 School of Science and Technology, and National Laboratory Astana, Nazarbayev University, Astana, Kazakhstan

**Keywords:** cytoskeleton, centrosome, microtubule dynamics, non-centrosomal microtubules

## Abstract

Microtubules in interphase fibroblast-like cells are thought to be organized in a radial array growing from a centrosome-based microtubule-organizing center (MTOC) to the cell edges. However, many morphogenetic processes require the asymmetry of the microtubules (MT) array. One of the possible mechanisms of this asymmetry could be the presence of non-centrosomal microtubules in different intracellular areas. To evaluate the role of centrosome-born and non-centrosomal microtubules in the organization of microtubule array in motile 3T3 fibroblasts, we have performed the high-throughput analysis of microtubule growth in different functional zones of the cell and distinguished three subpopulations of growing microtubules (centrosome-born, marginal and inner cytoplasmic).

Centrosome as an active microtubule-organizing center was absent in half of the cell population. However, these cells do not show any difference in microtubule growth pattern. In cells with active centrosome, it was constantly forming short (ephemeral) MTs, and ∼15–20 MT per minute grow outwards for a distance >1 µm. Almost no persistent growth of microtubules was observed in these cells with the average growth length of 5–6 µm and duration of growth periods within 30 s.

However, the number of growing ends increased towards cell margin, especially towards the active edges. We found the peripheral cytoplasmic foci of microtubule growth there. During recovery from nocodazole treatment microtubules started to grow around the centrosome in a normal way and independently in all the cell areas. Within 5 minutes microtubules continued to grow mainly near the cell edge. Thus, our data confirm the negligible role of centrosome as MTOC in 3T3 fibroblasts and propose a model of non-centrosomal microtubules as major players that create the cell asymmetry in the cells with a mesenchymal type of motility. We suggest that increased density of dynamic microtubules near the active lamellum could be supported by microtubule-based microtubule nucleation.

## 1. Introduction

Microtubules (MTs) are dynamic structurally polar cytoskeletal components that grow by addition of the GTP-loaded tubulin to their end. In eukaryotic cells, MTs are responsible for the intracellular organization, directed locomotion, and division. The centrosome is considered the major MT-nucleating center in the fibroblast-like cells [1]. However, growing body of evidence shows that this is oversimplification [2] and in many types of animal cells centrosome is not working as MTOC through interphase [3]. In our previous study we concluded that “in fibroblasts (1) the default behavior of free MTs in the cell interior is biased dynamic instability (i.e., random walk of the plus ends with significant positive drift); (2) MTs born at the centrosome show ‘dynamic instability’ type behavior with no boundary; (3) that the extended radial array is formed predominantly by MTs not associated with the centrosome” [4]. In the current study, we extended our analysis to describe MT array further and to determine what part of the MT array might be directly associated with the centrosome and what part is regulated by local properties of the cytoplasm.

It was suggested that MT growth is initiated by the centrosome working as a conveyor for making microtubules that are further released and continue their lives in the cytoplasm [5]. Later observations confirmed that MTs could detach from the centrosome [6]. However, this is a rare event [7],[8]. Growing body of evidence demonstrates that majority of MTs are functioning independently from the centrosome. Still, there is no quantitative data on the possible impact of the centrosome into the overall MT array. It was suggested that in some cells about 50% of MTs could originate from Golgi complex, while others are still thought to grow from the centrosome [9]. MT growth in many types of cells is not as persistent as originally proposed [10]–[12], however, the detailed comparison between duration of MT growth and cell radius is lacking. In the current report, we have analyzed the spatial distribution of growing MT ends and demonstrate that centrosome is responsible for less than 10% of the population of growing MTs. More than that, about 50% of the cultured 3T3 cells have no “visible” centrosome operating as MT organizing center. It means that in such cells centrosome is responsible for less than 1% of growing MTs. We also demonstrated that during recovery from nocodazole treatment centrosomes are not activated, and growth of MTs occurs predominantly near the cell margin.

## 2. Methods

### 2.1. Cell culture

NIH-3T3 fibroblasts were maintained in DMEM medium supplemented with 25 mM HEPES (PanEco, Russia), 10% fetal calf serum (PAA Laboratories, Austria), and 0.8 mg/mL gentamycin at 37 °C at 5% CO_2_. Cell suspension obtained with trypsin-EDTA (PanEco, Russia) was plated in 35 mm Petri dishes. The cell density was adjusted so that spreading cells did not contact each other.

### 2.2. Transfection

For visualization of alpha-tubulin, 3T3 fibroblasts were transfected with X-tremeGENE HP DNA transfection reagent according to manufacturer's protocol. Briefly, 1 µL of alpha-tubulin GFP plasmid and 2 µL of the agent were added to 100 µL of PBS, incubated for 20 minutes and then transferred into Petri dish with cells in 2 mL of DMEM medium, the observation started in 24 hours of incubation. The same protocol was applied for centrin (pEGFP-C1-centrin-1, Addgene # 72641). For visualization of MT growing ends, EB3-RFP gene was cloned into a pGPV lentiviral vector containing copGFP as a reporter and puromycin as drug selection marker (Eurogene, Russia). The lentiviral vector was transduced into HEK293 packaging cells (ATCC # CRL-11268) with X-tremeGENE HP DNA transfection reagent according to manufacturer's protocol, viral harvest was collected in 36 and 72 hours after transfection and then used for transduction of 3T3 cells.

### 2.3. Immunofluorescence

MT array was visualized with antibodies to α-tubulin (clone DM1A, eBioscience, U.S.A), Golgi complex was visualized by double staining with monoclonal antibodies to 58 K, Golgi protein (Sigma, U.S.A) and secondary Cy-2 antibodies (Sigma, U.S.A). Cells were fixed with 2.5% glutaraldehyde in PBS, pH 7.2, for 15 min at 4 °C, washed three times, permeabilized with 0.1% Triton–X-100 in PBS for 1 h at room temperature, washed three times with 1% sodium borohydrate, and stained with antibodies.

Fixed cells were imaged on Nikon Ti-E microscope under PlanApo × 60/1.4 objective (phase contrast) with CoolSnapHQ digital camera using filter sets for FITC and DAPI.

### 2.4. Inhibitory analysis

To fully depolymerize microtubule system cells were treated with nocodazole (Sigma) in the concentration of 13.2 × 10^−6^ М.

### 2.5. Microscopy

Live imaging was carried out on inverted Nikon Ti-E microscope with the PlanApo × 60/1.4 oil immersion objective at 36.5–37 °C, Hamamatsu Orca Flash 4.0 digital camera, driven by MicroManager software, was used for image recording, time intervals between frames were 2 seconds.

### 2.6. Subtraction analysis

We detected position of MT plus ends by subtraction of sequential images (I_n_-I_n+1_) in obtained time-lapse series as described previously [4],[13],[14].

### 2.7. Centrosome brightness measurement

The brightness of centrosome was defined as a number of EB3-RFP comets stated at the centrosomal area. The integrated density was measured in Fiji ImageJ for 5 average EB3-RFP comets (IDcom(1-5)) in each cell and integrated density of cytoplasm area (IDcyt(1-5)) of the same size located near EB3-RFP comets and containing no comets. Then we counted centrosome area integrated density visualized as a cluster of EB3-RFP at the z-stack slices (IDc(1-4)) We measured integrated density for cytoplasm area of the same size as centrosome was defined (IDo(1–4)) which was established as background for the measurement. As soon as centrosome was seen in 4–5 slices of z-stack, we summed integrated density measured at all slices where centrosome was seen, to have a number of comets located near the centrosome in volume.

The mean integrated density for comets was measured for each cell by the formula:

meanIDcom=(IDcom(n)−IDcyt(n))+(IDcom(n+1)−IDcyt(n+1))/n

Where n is the number of the measurement.

The summed integrated density for centrosome was measured for each cell by formula:

MeanIDc=((IDc(n)−Ido(n))+(IDc(n+1)−Ido(n+1))

Where n is the number of measurement.

To finally recalculate the brightness of centrosome we used the formula:

Brightness=meanIDc/meanIDcom

For each cell, the integrated brightness was calculated separately due to the differences in fluorescence intensities.

### 2.8. Measuring the density of growing tracks in cell interior and the MTOC activity of centrosome

The distribution of growing MT plus-ends along the cell interior was determined by counting EB3-comets in each of regions delineated by concentric circles placed at a distance of 5 µm from each other (for the cells with the radius 35 µm). MT plus-ends started in 2 µm of the cell edge were counted and analyzed as a separate group. EB3-RFP comets counts were normalized for each area and plotted as a function of distance along the cell radius (Figure S1).

We analyzed the distribution of growing MT plus-ends in eight cells expressing tubulin-GFP as a control. We measured growing MT ends as it was performed for cells with EB3-RFP comets, but excluded from the analysis MTs, growing at more than 20 µm from cell edge, as soon as MT network in this area was too dense to identify growing events precisely.

The frequency of MT nucleation at the centrosomal area was estimated from time series of images of cells expressing EB3-RFP by counting the EB3-RFP comets that started at centrosome area delineated the radius of 2 µm (the actual size of centrosome). We excluded from analysis the tracks that started at the centrosome and had the length less than 1 µm, as soon as they were considered the result of dynamic instability growth.

### 2.9. Analysis of microtubule dynamics

MT dynamics were measured by acquiring time-lapse series of images of the cells, expressing EB3-RFP, and manually tracking individual MT ends using Fiji ImageJ software. All the cells taken for analysis had the typical fibroblast morphology with large lamella at the leading edge of the cell and didn't significantly change their location and area during the observation time (100 seconds, 2 seconds per frame). During 100 seconds of observation cell area and shape descriptors didn't change significantly. Average cell area (N = 89) was 2116.0 ± 135.2 µm^2^ (mean ± SEM) and axis ratio was 3.89 ± 1.2.

Parameters of MT dynamics were determined by the length of growing periods (µm), growth rate (µm/min) and growth period lifetime (seconds). Using z-stacks (steps 0.5 µm, 0.5 seconds per frame, 10 frames) we analyzed the distribution of EB3-RFP comets around the centrosome area and observed barely few comets above and below it. Thus for the further analysis, we made time-lapse series at a single focal plane where the centrosome position is seen precisely. We divided MTs into three groups for the further analysis—MTs started at the centrosomal area (those that crossed the circle with 10 µm radius and centrosome in its center), free MTs in cytoplasm and MTs growing at the edge of the cell (MTs started at 2 µm area from cell margin).

To estimate the direction of MT growth in established previously cell areas we regarded plus-end growing track as a vector with the beginning at the first comet track and the end in the last comet of the track. The direction of MT growth was measured as an angle between growing MT vector and 0° vector. For centrosomal tracks, 0° vector was established as perpendicular started at the centrosome and directed to the cell edge. Free MTs and MTs at the cell edge were compared to perpendicular growing from geometrical center of the cell to the cell border. Thus, if MT was growing radially to the edge of the cell the angle was 0° and if it grew the opposite way the angle was 180°.

Statistics data were obtained with the Sigma Plot 3.0 software (Jandel Scientific, U.S.A), and data are presented as mean values with a standard error of the mean. Fluorescent images were processed using ImageJ and Adobe Photoshop (Adobe, U.S.A) software.

## 3. Results

### 3.1. General characterization of MT array in 3T3 cells

**Figure 1.**
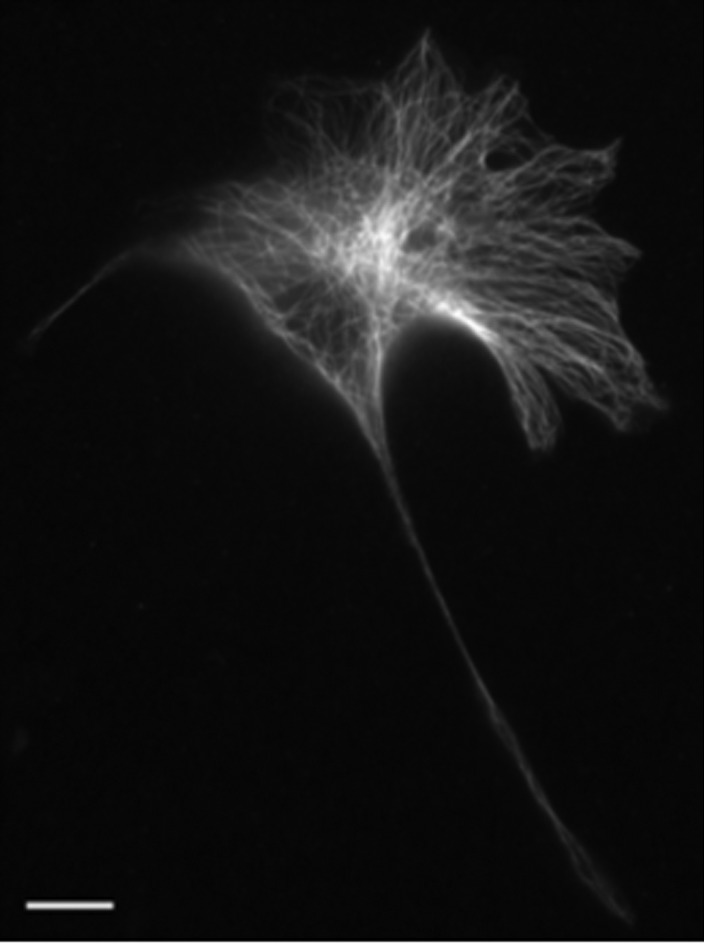
MT array in a representative 3T3 cell, immunostaining with antibodies to alpha-tubulin, MTs are organized in a radial pattern, bar 10 µm.

Tubulin staining showed that MTs in 3T3 cells were organized in a standard array with relatively high density around the nucleus and sparser array near the cell margins. In many cells MT array was organized as individual MTs running in all directions and in some other cells it was nearly perfectly radial, i.e., individual MTs were running along cell radius (Figure 1). In elongated cells, the main part of growing microtubules was oriented parallel to the longest axis.

**Figure 2.**
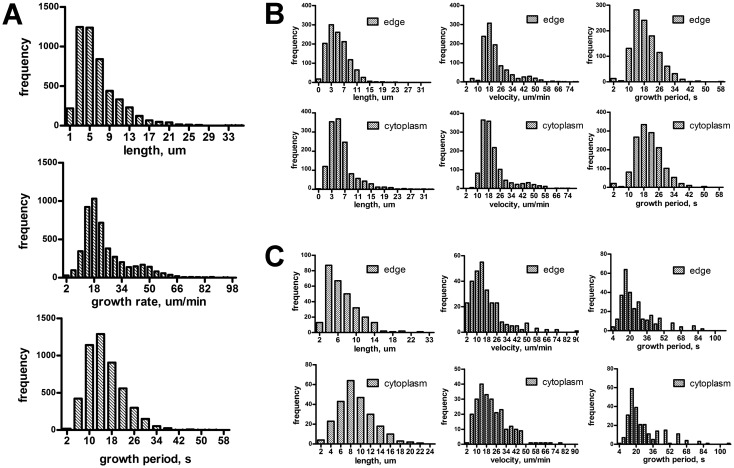
A: Distribution of dynamic parameters for the total MT population determined using EB3-RFP tracks in 89 cells. B: Distribution of dynamic parameters for the MTs in the inner cytoplasm (N = 444) and near the cell margin (N = 2194) measured by EB3-RFP tracks, 89 cells were analyzed. C: Distribution of dynamic parameters for the MTs in the inner cytoplasm (N = 245) and near the cell margin (N = 287) measured by GFP-tubulin tracks in 10 cells.

To describe dynamic parameters of MTs, we analyzed 4800 MT tracks in 89 EB3-RFP transduced 3T3 cells. The average length of track was 5.7 ± 5.5 µm (range from 1.2 to 59.1 µm), mean duration of growth was 17.3 ± 16.1 s (range from 6 to 72 s), the growth rate was 21.5 ± 19.4 µm/min (range from 3.3 to 29.5 µm/min) (Figure 2A). Notably, the frequency distribution of growth rate had the major peak on 18 µm/min and the minor peak on 46 µm/min, corresponding to a small subpopulation of the rapidly growing MTs. Only 158 MTs out of 4800 grew longer than 40 s and 169 MTs had the track length more than 10 µm that is still much shorter than 3T3 cell radius. Thus we observed relatively short growth periods of MTs, which is consistent with the previous studies [15]. The major pool of these short MT tracks in spread 3T3 cells grew in the cytoplasm or near the cell margin, whereas only a few of them started at the centrosome.

Previous studies established that MT growth rates detected using fluorescently-labeled + TIPs are faster than those measured using fluorescently labeled tubulin [16]. The difference is explained by slight pauses in MT growth that can be missed with the use of + TIPs as soon as they mark only MT plus ends. To prove the consistency of our results we evaluated the dynamic parameters of MTs labeled with GFP-tubulin in 8 3T3 cells. For cells expressing tubulin-GFP, growth rate was 20.7 ± 8.5 µm/min, MT length was 8.8 ± 3.6 µm, and the duration of growth was 25.6 ± 10.6 seconds.

According to the previous studies [11], MTs in the internal cytoplasm could be separated into two major groups by growth orientation-nucleated at the centrosome and free MTs (MTs growing nearly perpendicular to the cell radius with growing segments straight or slightly curved). To make the analysis of free MTs more explicit, we subdivided them into MTs growing at the cell margin (<2 µm from the margin) and MTs growing in the inner cytoplasm (starting at >5 µm from centrosome). MTs originated from the centrosomal area were analyzed separately.

### 3.2. Centrosome is a dense cluster of EB comets and is present only in a half of 3T3 cells

**Figure 3.**
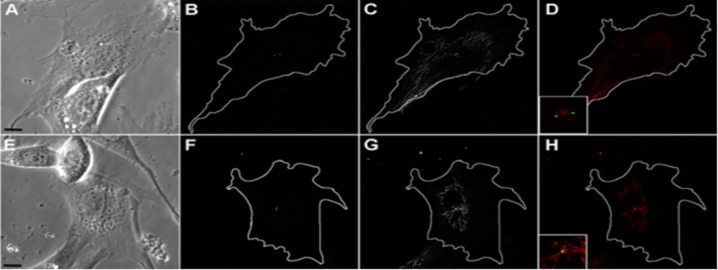
Centrosome as an active MTOC can be visualized as a dense cluster of EB3-RFP comets in half of 3T3 cells (27 of 43), bar 10 µm. A: Phase contrast image of cell 1. B: 3T3 cell (cell 1) transfected with centrin, centrosome can be seen as two bright spots. C: EB3-FRP transfected cell, MIP projection of EB3-RFP tracks for 100 seconds of time-lapse video. D: Merge, no EB3-plus ends is growing from the centrosome. E: Phase contrast image of cell 2. F: 3T3 cell (cell 2) transfected with centrin, centrosome can be visualized as two bright spots. G: EB3-FRP transfected cell, MIP projection of EB3-RFP tracks for 100 seconds of time-lapse video. H: Merge, one of the centrioles is functioning as MTOC.

In EB3-RFP transduced cells we defined the active centrosome (functioning as MTOC) as a dense cluster of comets in cell center with high values of integral brightness (corresponding to 32.9 ± 6.7 single comets) and a large number of short microtubules starting to grow from this area (18.0 ± 7.0 growth events per minute). Centrosome as a cluster of EB3-RFP growing events was observed on 4 subsequent optical sections. Thus the diameter of visualized centrosome along z-axis was not less than 1.5 µm. To evaluate the centrosome-nucleated MTs we analyzed 124 z-stacks collected with 0.5 µm increment to identify the presence of active centrosome. We found the active centrosome in 68 cells out from 124, while in the rest 56 we detected no centrosome functioning as MTOC. To confirm this observation, we analyzed 43 cells simultaneously labeled with EB3-RFP and centrin as a conventional centrosomal marker. In 27 cells out of 43, we observed active centrosome with integral brightness corresponding to 30.2 ± 8.7 single EB3-RFP comets and having nucleation rate 14.7 ± 3.6 growing comets per minute. In all these cells, centrin was detected as one or two bright spots with a mean area 1.2 ± 0.4 µm in the center of EB3-RFP cluster of growing plus-ends (Figure 3A–D).

In 16 cells we also detected one or two bright spots of centrin (depending on the phase of the cell cycle) of the same size, but all events of MT growth in these cells were scattered alongside the cytoplasm with no focused clusters in the perinuclear area (Figure 3E–H). Thus, about the half of 3T3 cells in interphase contain the active centrosome functioning as MTOC and the activity of the centrosome does not depend on the phase of the cell cycle.

### 3.3. MTs originated from centrosome represent a small part of all MT population

In cells with centrosome functioning as an active MTOC, we observed 1560 tracks originating at the centrosome with average length 5.3 ± 2.7 µm and nucleation rate 18.0 ± 7.0 µm/min. Out of them, 562 continued to grow for not more than 12 s, 74 MTs continued their growth for 15–40 s and only 5 grew persistently for a longer period. Analysis of the maximal intensity projection series showed that only in few cells we could trace long tracks radiating from the centrosome towards cell periphery (Figure S2). Moreover, analysis of z-stacks showed that MT cluster growing from centrosome and growing MTs near the cell margin were visualized in different optical sections and Z-distance between them was not less than 3 µm (data not shown). Thus, most of the MTs started at the centrosome didn't reach the cell margin.

### 3.4. Spatial organization of MT growth in the cytoplasm

For EB3-RFP expressing cells, the duration of growth of free MTs at the cell margin (N = 2194) was 14.0 ± 0.3 s with median growth rate 21.0 ± 0.7 µm/min. The length distribution was gamma-shaped (γ = 2.67), with a median of 5.3 µm. For MTs in the inner cytoplasm (N = 444), the median length and duration of growth were slightly higher (5.4 ± 0.1 µm and 16.0 ± 0.1 s), the differences between these values for MTs at the cell edge and in the inner cytoplasm were statistically significant (Mann-Whitney test, *p* < 0.05). For tubulin-labeled cells the length for MTs in the inner cytoplasm (N = 245) was 8.6 ± 0.2 µm compared to 6.3 ± 0.2 µm for MTs at the cell margin (N = 287), the differences between the values were also statistically significant (Mann-Whitney test, *p* < 0.001). The distribution shapes of the histograms for EB3-RFP and tubulin-labeled cells were similar (Figure 2B compared to Figure 2C).

Next, we built vector histograms of growth angle distribution for subpopulations of MTs: 1) MTs, growing from the centrosome; 2) MTs in the inner cytoplasm; 3) MTs near the cell margin. MTs started at the centrosome demonstrated isotropic growth pattern, most of MTs grew rectilinearly and didn't bend. The average angle of growth for centrosomal MTs was 156° ± 100°.

**Figure 4.**
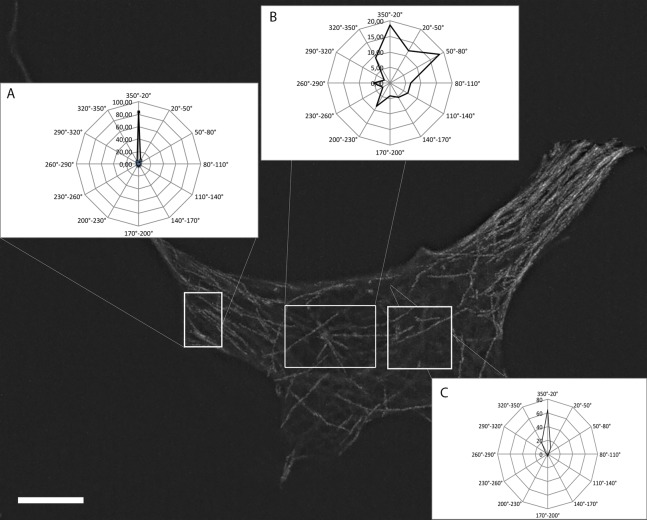
Vector histograms of growth angle distribution for subpopulations of MTs near the centrosome, in the inner cytoplasm and near the cell margin. A: Angle distribution of MT growth in the cell margin. B: Angle distribution for MTs in the centrosomal area. C: Angle distribution for MTs in the inner cytoplasm.

MTs growing in inner cytoplasm independently from centrosome (more than 10 µm from centrosome) were organized in a more chaotic pattern. 20 MTs out of 1087 grew oppositely to the leading edge, while the main part of the population (706 MTs out of 1087) was oriented toward the leading edge. The average angle of growth was 102° ± 78° (Figure 4).

In contrast, only 8 tracks out of 1148 near the cell margin (less than 10 µm from the cell edge) were growing towards the cell interior, while 959 MTs out of 1148 grew towards to the edge of the cell. The average angle of growth for this subpopulation was 152 ± 110°. The same organization of MT growth orientation was observed in cells with active or inactive centrosomes.

**Figure 5.**
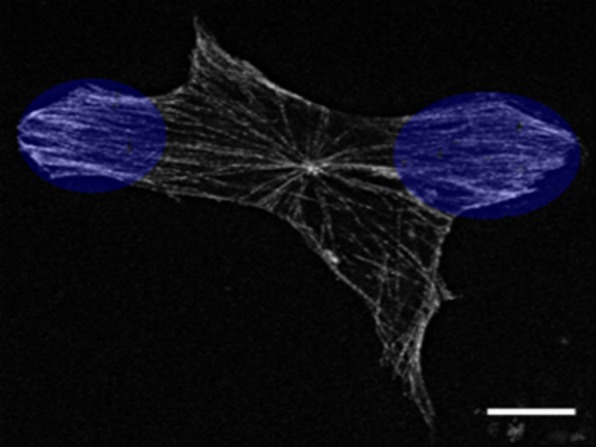
MIP projection of 100 seconds for EB3-RFP tracks in the 3T3 cell, the highest density of growing MTs is present in some parts of the cell, yet always near the plasma membrane.

To analyze the spatial distribution of non-centrosomal MTs, we built maximal intensity projection tracks. These tracks were mainly straight or slightly curled (Figure 5). Typically, curled tracks were present near the cell edges, and the highest density of growing MTs was always present near the plasma membrane. We named these parts of cell edge “active” regarding initiating growth of numerous MTs.

To evaluate the difference in the numbers of growing MTs between cell areas we performed the following measurement: We measured the density of EB3-RFP plus-ends for 300 comets in 7 cells. We selected the equal areas near the active centrosome (at a distance 2–4 µm from the bright cluster of comets), in the cell interior and near two types of the cell margin, one with active lamellae and the other which was stable (Figure S3).

**Figure 6.**
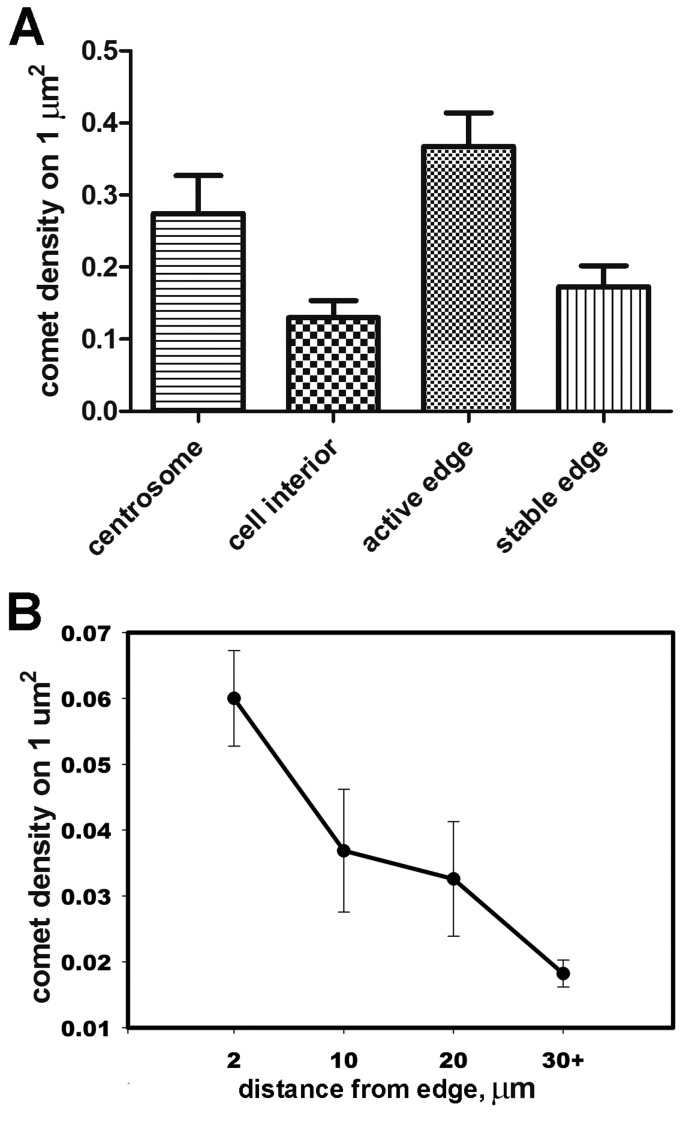
A: The density of EB3-RFP plus ends per 1 µm^2^ in different cell areas. Data are presented as mean ± SEM, the differences between densities for active edge and stable edge and between the centrosome and cell interior are statistically significant (453 comets in 7 cells were analyzed, *p* = 0.005, Mann-Whitney test). B: Distribution of the density of growing MT plus-ends versus length in the elongated cells, the dataset consisted of 256 comets in 10 elongated 3T3 cells.

Notably, the distribution of the EB3-RFP plus-ends was not uniform for the leading edge with active lamellae and stable edges. The highest density of EB3-RFP comets was found for the active edge (at the distance of 2 µm from the cell margin)—0.34 ± 0.05 comets per 1 µm^2^, which was twice higher than comet density at the stable edge (the difference was statistically significant, *p* = 0.005, Mann-Whitney test) (Figure 6A). More detailed analysis of 256 comets in 10 elongated cells showed that the density of MT tracks increases linearly from cell interior towards the edge (Figure 6B). The density of growing ends near the stable edges was similar comets density in the inner cytoplasm as described elsewhere [13].

Hence, the population of the non-centrosomal MTs can be subdivided into two independent groups of MTs at the cell margin, and MTs in the inner cytoplasm, MTs in these two subgroups have different dynamic parameters. The differences in angle distributions and the density of growing plus ends near the cell edge, and in the cell interior gave us an idea to look for the organization patterns of cytoplasmic MTs.

### 3.5. MTs can originate from alternative non-centrosomal MTOC in the cytoplasm

In order to find if there were special “hot spots” in cytoplasm with high density of growing plus-ends, we divided cytoplasm into areas with 2 µm diameter and chose those that fit the following criteria: (1) we observed curving MTs in this regions; (2) the number of tracks starting in such region was more than average over the cytoplasm (3 and more growth events per minute).

As a result, we found 113 areas in the cytoplasm of 89 cells that corresponded to the parameters mentioned above. Average number of MTs starting to grow at these points was 6.2 ± 0.9 events per minute while in other areas of the cell with the same size we observed only 2.0 ± 0.7 growing MTs per minute. Those cytoplasmic “foci” were detected both in cells with the active or inactive centrosome. The main part of these “hot spots” of MTs growth was located at the cell periphery, at a distance 5.6 ± 2.5 µm from the cell margin. The number of “foci” varied from 1 to 3 per cell, but all the cells contained at least one (Figure 8). We observed only one such “hot spot” in 41 cells, 21 cells had two of them, and 10 cells had three.

Dynamic parameters of MT growth for the cells with one cytoplasmic focus were slightly higher (Table 1). We also compared dynamic parameters of microtubules originated from the centrosome and the cytoplasmic areas of MT growth. The dynamic parameters for MTs growing at cytoplasmic foci were similar to the dynamic parameters of centrosomal MTs.

**Table 1.** Dynamic parameters of MTs in cells with one or two cytoplasmic foci of MT growth (data are presented as median ± SEM).

	1 focus per cell	2 foci per cell
Number of cells	41	21
Number of tracks	1348	1202
Length, µm	8.22 ± 0.51	6.21 ± 0.43
Duration, s	16.54 ± 0.22	15.82 ± 0.27
Growth rate, µm/min	30.02 ± 0.44	24.81 ± 0.49

**Figure 7.**
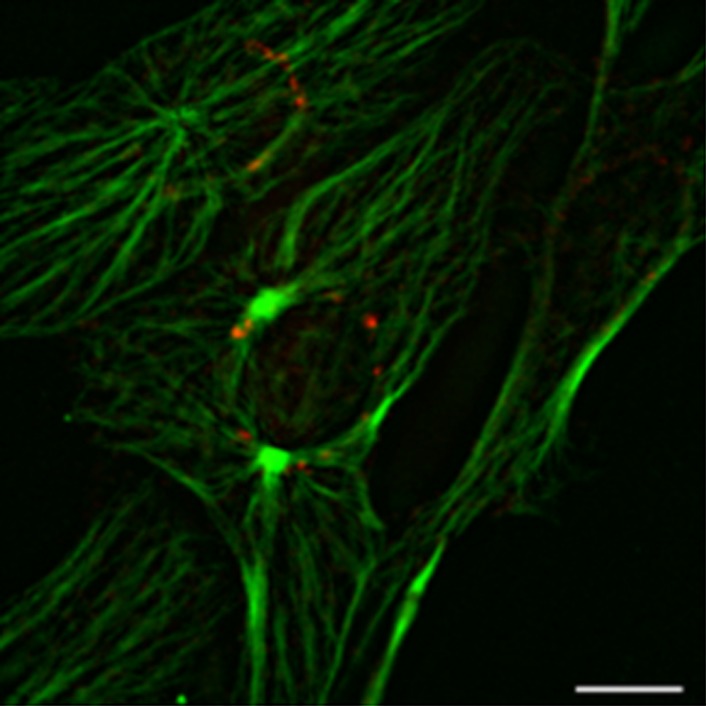
3T3 cell co-stained with the antibodies to Golgi 58K protein (red) and alpha-tubulin (green). Golgi apparatus is not co-localized with the clusters of MT growth in the cytoplasm, bar 10 µm.

**Figure 8.**
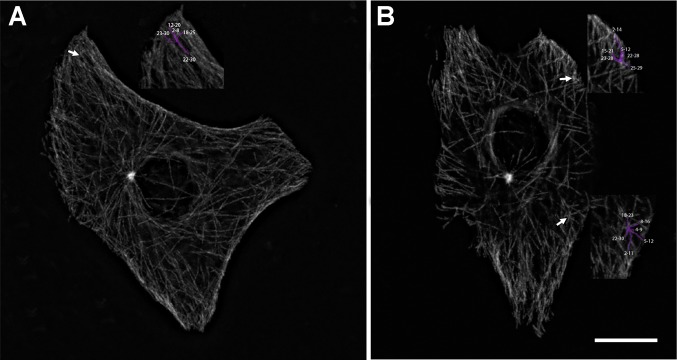
Cytoplasmic foci of MT growth as seen on maximal intensity projection images, bar 10 µm. A: Cell with one cytoplasmic focus; B: a cell with two cytoplasmic foci, tracks growing from each focus are visualized. Cell nuclei are visible in the center of each cell as an oval area with a low level of EB comets.

Previous studies [17],[18] showed that such cytoplasmic areas of MT growth might be associated with Golgi apparatus in epithelial cells. To elucidate the role of Golgi apparatus as a cytoplasmic MTOC in 3T3 cells, we performed the co-staining of 3T3 fibroblast with antibodies to the 58K protein, a conventional Golgi marker, and alpha-tubulin (Figure 7). We observed a very low level of co-localization between Golgi apparatus and the cytoplasmic clusters of MT growth, as most of the Golgi membranes were located in a perinuclear area, while the cytoplasmic foci of MT growth not associated with centrosome were usually localized near the cell edge and far from the nucleus (Figure 8).

Thus, cytoplasmic foci of MT growth in 3T3 cells are not associated with Golgi apparatus.

### 3.6. Microtubule system recovery after nocodazole treatment starts in the inner cytoplasm and at the centrosome

To test whether cytoplasmic MTs foci are responsible for MT array organization, we described the spatial patterns of MT recovery after complete MT depolymerization. We treated 3T3 cells (N = 350), transfected with tubulin-GFP, with 4 µM nocodazole for 1 hour and then washed it out. Cells were fixed at 5 and 60 minutes after nocodazole withdrawal.

MTs started to grow rapidly all over cytoplasm in 2 minutes after nocodazole withdrawal. In these cells, tubulin could be visualized as a bright cluster of short MTs, organized in the radial matter. MT clusters were localized near the nucleus and could be considered as an active centrosome, which was detected in 270 cells out of 350. In 80 cells only diffuse growth of MTs in the cytoplasm was observed.

To confirm that Golgi apparatus is still not associated with cytoplasmic MT growth in experimental conditions, we co-stained 3T3 cells after nocodazole treatment with antibodies to 58K and alpha-tubulin. MTs depolymerization led to Golgi apparatus dispersal. The first growing MTs started at the centrosome were observed at the third minute after nocodazole washout. In half of the population (65 cells out of 115) we found additional growing asters of tubulin in the cytoplasm. They were located at the cell periphery (at a distance less than 5 µm from the cell margin) and were not co-localized with Golgi apparatus, the average area for Golgi apparatus was 5.6 ± 1.8 µm^2^ that is consistent to control cells (Figure S4). One hour after nocodazole withdrawal there were no additional clusters in the cytoplasm, and the dense MT network was observed near the centrosome. Most of the cells (98 out of 115) had a visible centrosome in perinuclear area.

To characterize dynamics of MT recovery after nocodazole treatment, we observed 30 3T3 cells with stable expression of EB3-RFP under the same conditions. In the presence of nocodazole, there were no EB3-RFP comets in the cytoplasm, but some cells still had bright centrosome as a 2 µm diameter cluster with EB3-RFP comets in the cell center.

**Figure 9.**
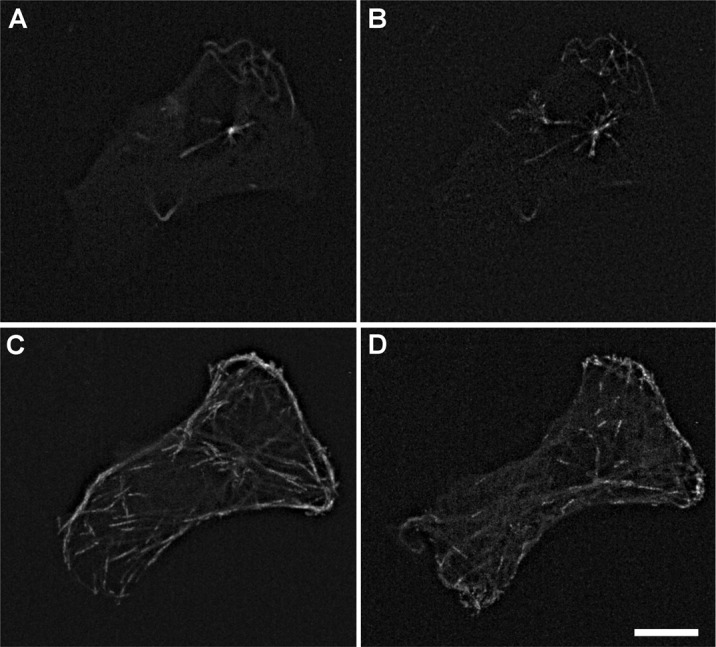
MT array recovery after 1 hour of incubation with nocodazole. A: MIP projection of 60 seconds of EB3-RFP transfected cell in the presence of nocodazole, no growing comet in the cytoplasm is observed, but the centrosome still can be seen as a bright spot. B: Same cell, MIP projection of EB3-RFP tracks for 60 seconds of time-lapse video at 1 minute after nocodazole withdrawal, growing comets are seen in cytoplasm area and across cytoplasm. C: Same cell, MIP projection of EB3-RFP tracks for 60 seconds of time-lapse video 5 minutes after nocodazole washout. D: Same cell, MIP projection of EB3-RFP tracks for 60 seconds of time-lapse video at 60 minutes after nocodazole withdrawal.

EB3-RFP comets appeared immediately all over cytoplasm at first minutes after nocodazole withdrawal. Although most of the cells (28 out of 30) contained active centrosome, some microtubules started to grow in inner cytoplasm far from centrosomal MTOC (Figure 9). The clusters of cytoplasmic growth were smaller in size than the active centrosome (1.6 ± 0.5 µm diameter) and located at 5 to 15 µm from the cell nucleus. The frequency of growth events at first minutes after nocodazole withdrawal was 421 ± 114 events per minute that is 1.5 times lower than in untreated cells. In 60 minutes the number of growth events increased to a normal rate and was counted as 674 ± 174 events per minute. The duration of MT growth periods at first minutes after nocodazole withdrawal was less than after 60 minutes (11.3 ± 3.3 versus 15.0 ± 5.2 seconds). After 1-hour centrosome in most cells was still active. Nucleation rate for active centrosome was similar to untreated cells (21.0 ± 4.8 vs. 18.0 ± 7.0 growth events per minute, respectively).

## 4. Discussion

In cells with a mesenchymal type of motility, MT array is organized in a radial pattern near the centrosome and is relatively chaotic near the cytoplasm [11]. In our work, we tracked almost 5000 MTs in different functional zones of the cell. All the analyzed MT tracks demonstrated relatively short length in comparison to cell radius despite their location in the inner cytoplasm. Almost none of MTs proceeded directly from centrosome to the edge of the cell, which imposes a possibility to distinguish centrosomal MTs from MTs in the cell interior and MTs near the cell edge.

### 4.1. The growth of MTs is nearly uniform in the entire cytoplasm

The overall distribution of growth length and rates in 3T3 fibroblasts shows a remarkable feature-while the length distribution is gamma-shaped, distribution of growth rates shows the presence of two peaks corresponding to slow and fast MTs. This is similar to the observations on endothelial cells where the first peak appeared 18–20 µm/min and a second maximum—at 45–50 µm/min [19]. The same correlation was observed in another study, where the instantaneous rate of microtubule growth was similar for the leading edge of polarized epithelial cells and fibroblasts: In LLCPK1 cells the average growth rates were 17.9 ± 7.7 µm/min, in CHO cells—16.0 ± 6.8 µm/min [20]. Similar values of MTs growth rates (16–17 µm/min) in the internal cytoplasm for CHO cells were obtained in the case of plus-end tracking proteins labeling [12].

However, the detailed analysis showed that in endothelial cells slow and fast MTs are growing exclusively from the centrosome, while in 3T3 cells these two groups are growing in the entire cytoplasm, with similar distributions for the cytosolic and marginal subpopulations.

Histograms of MT growth in the internal part of the cytoplasm and near the cell margin demonstrated that the main difference is in the length of tracks, which is significantly reduced near the cell edge. This happens along with the decrease of instantaneous rate, but not the duration of growth periods.

At the same time, the orientation of growing MTs in 3T3 cells has the same pattern as previously observed [11]. While MTs near the cell margin are oriented mostly towards the cell edge, free MTs in inner cytoplasm grow more chaotically which indicates that these two areas of the cell are regulated independently. Centrosomal MTs grow slightly isotropically, but most of MTs are oriented to the cell margin, possibly to maintain transport of growing MTs to the cell edge and to provide Golgi complex polarity.

### 4.2. Nucleation activity of the centrosome is not necessary for the organization of MT array in the interphase cells

Previous studies have already shown that long MTs nucleated at the centrosome in fibroblasts is a rare event [7],[8]. Centrosome constantly nucleates a significant amount of short MTs, which were described previously using electron microscopy [5],[21],[22] and with live imaging [4]. These ephemeral MTs are supposed to be supported by the oscillation of MT plus-ends in this area and the low frequency of rescue events for centrosomal MTs. Previous studies showed that frequency of rescues is modulated by several proteins, like CLIP-170 [12], EB proteins [23],[24], sentin [24]. Centrosome visualized with EB3-RFP looks like a bright cluster of EB3-RFP comets, which is consistent with the idea of the presence of a large number of short MTs in this area. We measured the brightness for active centrosome as 32.9 EB3-RFP comets, which means, that approximately 32 ephemeral MTs are generated in this area at the same time, a few of them could be somehow stabilized and become long MTs. The size of centrosome visualized with EB3-RFP on z-stacks was comparable to previous studies [4]. Recently the direct evidence on the ability of centrosomes to concentrate tubulin several-fold (about four times) against the cytosol was obtained [25]. So far, it is not a surprise that short microtubules can polymerize around the centrosome when microtubule polymerization in the cytoplasm is inhibited [5].

### 4.3. The active centrosome is not necessary for interphase cell functioning

In cells with non-centrosomal MT arrays, centrosome could serve as the main nucleation site of new tubulin polymers in interphase [26] and also contributes to cell cycle progression and cell abscission at telophase [27]. As soon as gamma-tubulin is the crucial player in MT nucleation, it is supposed to be found in non-centrosomal MTOC. Cells depleted with gamma-tubulin showed a delay in the dynamics of MT regrowth after full depolymerization by cold [28]. After the full recovery MT dynamics was similar to untreated cells. The same data was obtained for U2OS cells [29]. Our experimental data indicate that during recovery after nocodazole withdrawal MTs immediately start growing at the centrosome as well as in the entire cytoplasm. During recovery, the number of MTs growing from the centrosome does not exceed the values of the untreated cells. Non-centrosomal foci of MT growth appear after nocodazole treatment and disappear later. The similar foci in the cytoplasm, which are independent of the centrosome, were observed throughout S2 Drosophila cells during the cold recovery period [28],[30]. During the recovery, the cytoplasmic foci formed an extensive interconnecting network. Accumulation of EB3-RFP plus ends around the centrosome remained for a long time under nocodazole treatment (up to 20 h); however, the average intensity was ∼1/2 of that in control cells with the active centrosome.

As in the previous studies, we found that centrosome nucleates 15–20 MTs per minute. This raises a question, whether centrosome-born MTs generate a substantial part of the overall MT array?

Detailed analysis of the fate of MTs started growing from the centrosome in 3T3 cells showed that usually centrosome-born MTs do not continue growth after first pausing or catastrophe onset, instead undergo continuous shortening and often down to the zero-length [4].

Here we extended these observations and showed that in many cells (>50%) there is no visible activity of the centrosome, however, the entire MT array looks quite similar and the number of growing MT ends appearing in the cytoplasm per minute is 6.2 ± 0.9. Based on these data and taking into account that the density of MTs along ell radius does not decrease in the hyperbolic (y = a/x) curve that is expected in the case of the presence of long MTs running from the centrosome towards cell margin [1] we assume that MTs growing near the cell edge are independent of the centrosome.

### 4.4. Elongated cells often have asymmetric MT array

In elongated cells i.e., in fibroblasts and other cells with a mesenchymal type of motility MT array is polarized and highly asymmetric with most of MTs oriented to the direction of movement. This provides the non-uniform distribution of the signaling molecules [31] facilitating the actin reorganization, formation of adhesions [32] and vesicular traffic in the leading edge [33]. However, even in the polarized cells MTs nucleated at the centrosome initially form a radial array [20]. Concerning the average length of centrosomal MTs in 3T3 fibroblasts, we can assume the existence of non-centrosomal MTs that provide a major contribution to the asymmetry of the MT array. Another evidence for the negligible role of the centrosome in the organization of the MT array near the cell margin comes from the significantly decreased rate of nucleation of MTs from the centrosome in the cells at the edge of experimental wound [20] when the overall number of growth ends in the cell obviously increases (see Figure 2 in the aforementioned paper).

### 4.5. Non-uniform organization of the MT array in the leading and stable edges

The results of our experiments indicated that the difference in MT plus-ends distribution throughout the cell could be explained by changes in the parameters of the dynamic instability at different intracellular regions. Our data show that the highest density of growing plus ends is near the active cell edge where MTs are characterized by a high frequency of rescues [8],[34]. It could be provided by the activation of protein factors, promoting MT assembly, such as MAP4 [35], XMAP215 [36], or EB1 [23]. As a result, the catastrophe frequency decreases and MTs at the cell edge are highly dynamic, which could be important for interactions between MTs and cell adhesions at the cell edge. In the current study, we have also demonstrated a statistically significant difference in the densities of MT growing ends for the leading edge with active lamella and the stable edges.

Salaycik, et al. [20] showed that the average number of growing microtubules per µm^2^ is similar for leading and trailing edges and for cells containing one or two centrosomes. Our observations, however, show that in 3T3 cells there is a significant difference between active and stable edges in polarized randomly migrating cells whether or not these cells are elongated. It is interesting to notice that in poorly polarized CHO and LLCPK cells with relatively wide active edge growth of MTs from the centrosome is isotropic, while in the elongated 3T3 cells we found it anisotropic with dominating directions along the cell axis.

### 4.6. Alternative MTOC in the cytoplasm are located mostly at the cell edge

Our experimental data revealed the irregular distribution of growing plus-ends across the cell interior, with the highest density near the cell margin along with significantly low nucleation activity of centrosome. Thus we assumed the presence of compact domains in the cytoplasm, especially near the cell margin to provide MT nucleation and cell polarity in the interphase cells. Previous studies proved that several cell structures could act as a non-centrosomal MTOC. For instance, polarized non-centrosomal MTs accumulated alongside the apical cortex can be found in epithelial cells [37], around the nuclear envelope and Golgi apparatus in muscle cells [38],[39] and in axons and dendrites of mature neurons [40].

We observed the small clusters of MT growth in the cytoplasm of the 3T3 cells, where the number of growing MTs was 3 times more than the average values for cell interior. In all the cells, the localization of these clusters was not more than 5 µm from the cell margin. Accumulating body of evidence indicates that γ-TuRC can be involved in the nucleation of non-centrosomal MTs and/or anchor their minus ends in various types of cells, including neurons, epithelial cells and muscle cells [41]. The other candidate proteins that can serve as nucleation sites of the non-centrosomal MTs are the minus-end proteins. Previous studies indicated that CAMSAP2 and CAMSAP3 are crucial for maintaining and stabilizing non-centrosomal MTs in epithelial cells [17]. In RPE, HeLa, CHO and 3T3 cells CAMSAP2 anchors MT minus ends, slowing the rate of minus-end depolymerization and increasing the rate of microtubule plus end growth [42]. CAMSAP proteins in epithelial cells are clustered in 1.3 ± 0.6 µm^2^ size complexes in the cytoplasm [17] that is consistent with the size of cytoplasmic “foci” that we observed.

Recently augmin complex was described as one of the potent factors for MT polymerization on the existing MTs [43]. The current model [44] postulates that the augmin complex binds to MT walls and recruits the γ-TuRC through NEDD1 or other proteins to initiate new MT nucleation mainly along existing MTs [45],[46], or in an aster-like fashion [47]. We suggest that augmin complexes also work in the interphase cells; however, their activity is less evident. Peripheral foci of MT nucleation in the interphase 3T3 cells might represent such kind of augmin activity. These foci are always present near the cell margin and in the areas of maximal MT growth activity (i.e., near the active edges). The phenomenon of the stabilization of MTs in an augmin-like manner can explain the “bundles” of the parallel and highly dynamic MTs running alongside the cell margin.
