# Supplementary material for: Non-centrosomal MTs play a crucial role in organization of MT array in interphase fibroblasts
Source: AIMS Genet. 2018 Mar 28;5(2):141–60. doi: 10.3934/genet.2018.2.141 (PMC6698575; doi:10.3934/genet.2018.2.141)
Supplement: Supplementary file 1 [file genetics-05-02-141-s01.pdf]

AIMS Genetics

http://www.aimspress.com/journal/Genetics

AIMS Genetics, 5(2): 141–160.
DOI: 10.3934/genet.2018.2.141
Received: 05 December 2017
Accepted: 15 March 2018
Published: 28 March 2018

Research article

Non-centrosomal MTs play a crucial role in organization of MT array in interphase fibroblasts

**Yekaterina Zvorykina[1], Anna Tvorogova[2], Aleena Gladkikh[1] and Ivan Vorobjev[1,2,3,]*

[1] Biology Department, M.V. Lomonosov Moscow State University, Moscow, Russia
[2] A.N. Belozersky Institute of Physico-Chemical Biology, M.V. Lomonosov Moscow State University, Moscow, Russia
[3] School of Science and Technology, and National Laboratory Astana, Nazarbayev University, Astana, Kazakhstan

* **Correspondence:** Email: ivan.vorobyev@nu.edu.kz; Tel: +79166421053.

Supplementary

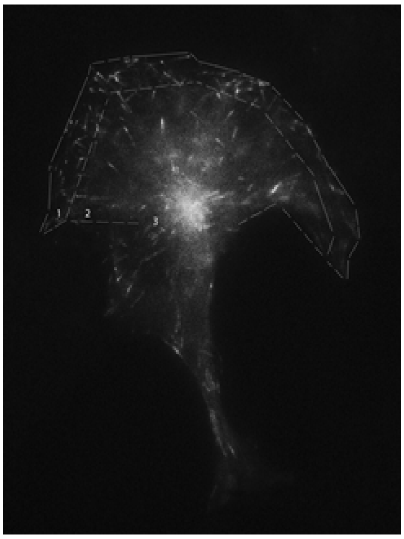

Figure S1. Assigned areas of the cell, 1: cell margin; 2: inner cytoplasm; 3: centrosome.

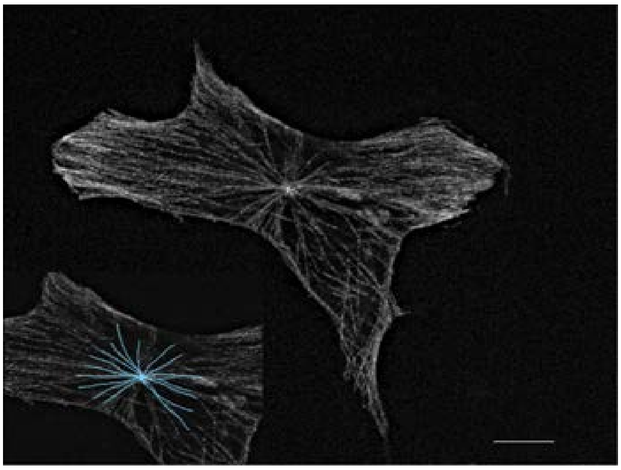

Figure S2. Maximal intensity projection of the 3T3 cell with stable expression of EB3-RFP (100 seconds of time-lapse video). Only a few of long tracks grow directly from the centrosome, bar 10 µm.

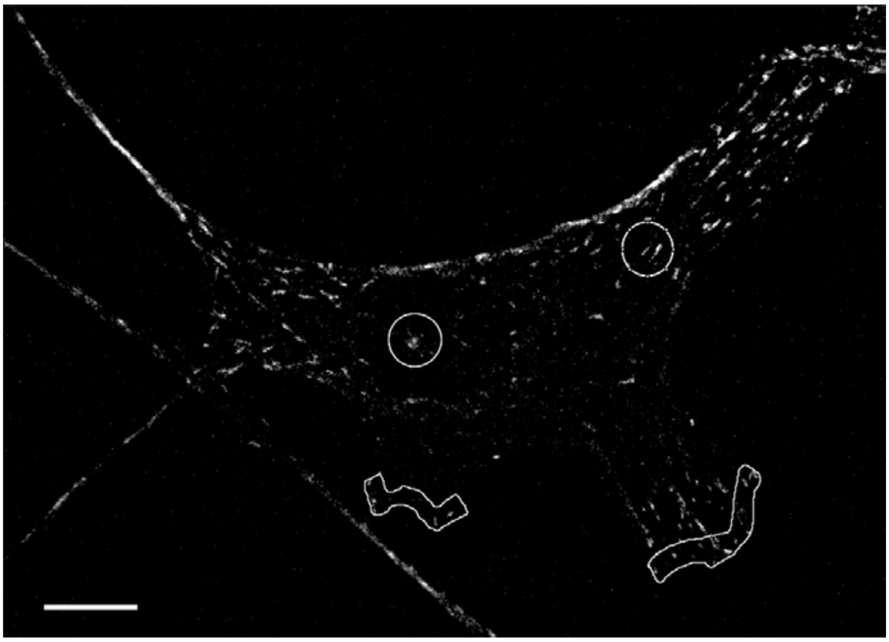

Figure S3. Measurement of EB3-RFP plus-ends density. Equal areas were subtracted in the cell interior, near the centrosome (not considering the centrosome area), near the cell margin with active lamella and near the stable edge (at a distance 0–2 µm from the cell margin), bar 10 µm.

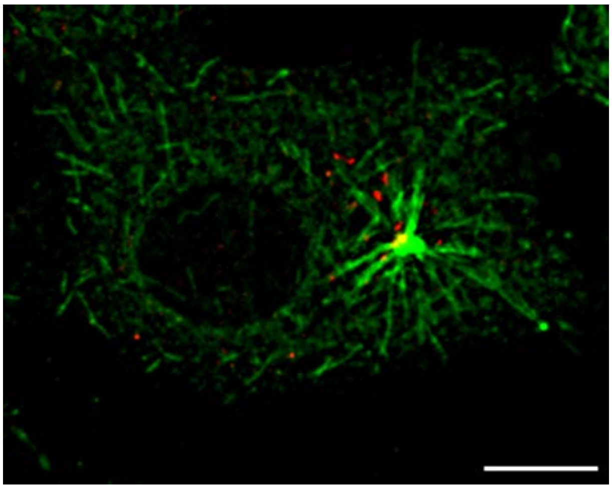

Figure S4. Co-staining for Golgi (red) and alpha-tubulin (green) in a cell after 1 hour of nocodazole withdrawal, bar 10 µm. Small clusters of MT growth are not co-localised

AIMS Press

© 2018 the Author(s), licensee AIMS Press. This is an open access article distributed under the terms of the Creative Commons Attribution License (http://creativecommons.org/licenses/by/4.0)
